# Needs assessment of community health workers to enhance efficient delivery of their services for community case management of malaria in Kenya

**DOI:** 10.1186/s12936-021-03640-2

**Published:** 2021-02-18

**Authors:** Michelle D. S. Boakye, Collins J. Owek, Elizabeth Oluoch, Sefa Bonsu Atakora, Juddy Wachira, Yaw A. Afrane

**Affiliations:** 1 College of Nursing, The Pennsylvania State University, University park, PA USA; 2 Centre for the Study of Adolescence, Nairobi, Kenya; 3 Mild May International, Kisumu, Kenya; 4 Department of Medical Microbiology, College of Health Sciences, University of Ghana, Accra, Ghana; 5 Department of Behavioral Sciences, School of Medicine, Moi University, Eldoret, Kenya

**Keywords:** Community Case Management of Malaria, Community Health Workers, Needs assessment

## Abstract

**Background:**

Malaria continues to be the leading cause of morbidity and mortality in Africa. Community Case Management of malaria (CCMm) which is undertaken by engaging Community Health Workers (CHWs) to effectively address management of malaria cases in some endemic communities was explored in this study. The aim was to assess the needs of CHWs that would help sustain and retain their services to enhance the efficient delivery of CCMm.

**Methods:**

Using semi-structured questionnaires, data on the needs of CHWs was gathered through a qualitative study consisting of in-depth interviews and focus group discussions (FGDs) conducted among study participants in five districts in western Kenya. The study participants comprised of 100 CHWs, 100 mothers of children under five years and 25 key informants made up of public health officers and clinicians involved in the CCMm. The interviews were conducted in English and Swahili or Dholuo, the local language. The recorded audio interviews were transcribed later. The analysis was done using NVivo version 7 software and transcripts were coded after which themes related to the objectives of the study were identified.

**Results:**

All the study participants recognized the need to train and update CHWs on their work as well as remunerating them for their services to enhance efficient delivery of services. The CHWs on their part perceived the provision of gloves, rapid diagnostic test kits (RDTs), lancets, cotton wool and ethanol, bins (to dispose of RDTs and lancets), together with drugs for treating clients as the essential needs to undertake CCMm in the communities. Other logistical needs and incentives mentioned by CHWs and key informants for the successful delivery of CCMm included: gumboots, raincoats, torch lights, mobile phones, means of transportation (bicycles and motorbikes), uniforms and ID cards for identification.

**Conclusions:**

CHWs would perform tasks better and their services retained for a sustainable CCMm if: properly incentivized; offered refresher trainings (and updates) on malaria; and equipped with the requisite tools identified in this study.

## Background

There are a number of ongoing malaria intervention programs in Africa, yet, malaria is reported to be the leading cause of morbidity and mortality in this region with 93 % of the 228 million malaria cases reported worldwide occurring in this region in 2018. Out of the 405,000 estimated global malaria deaths that year, 94 % occurred in Africa [1]. Of all the age groups, children under the age of five are considered the most vulnerable to malaria as 67 % of malaria mortalities occurred in that group [1]. Severe malaria among children under five is a common occurrence in western Kenya [2].

Prompt and accurate diagnosis of malaria together with appropriate treatment is key in malaria case management [3]. In Kenya, Community Case Management of malaria (CCMm) is undertaken by community health workers (CHWs) in malaria endemic regions in an effort to combat the burden of the disease [3]. CHWs are health volunteers selected from their communities and trained to work within villages [4]. In CCMm, CHWs diagnose malaria with rapid diagnostic tests (RDTs) and treat positive cases with artemisinin-based combination therapy (ACT) [5] thus, enhancing access to health care in these communities [6].

Studies have reported the effectiveness of CHWs in reducing child mortalities associated with malaria in several countries [3, 7, 8]. However, some of the previous studies identified challenges that could undermine the sustainability of CCMm [9–11]. These include: the lack of basic supplies and equipment to undertake assigned work; low level of support; and non-recognition of their services from clinicians [11]. The services of CHWs are crucial to the success of the CCMm and it is important to identify all gaps in relation to their needs so as to create an environment that would commit them to maintain their dedication to the programme. It is against this background that this qualitative study was conducted on the needs of CHWs that would help sustain and retain them to enhance efficient delivery of services for CCMm.

## Methods

### Study sites

The study was conducted in five districts in western Kenya where CHWs are undertaking CCMm. These include: Kisumu West, Nyando and Muhooni sub-counties that are found in the malaria endemic lowland regions; as well as Kisii and Kenyenya sub-counties found in the epidemic-prone highlands of western Kenya [12].

### Study participants

The study participants were made up of mothers/guardians of children who were less than five years and had received services from CHWs in the previous year. CHWs were selected from Community Units (CU) in the study sites (where CCMm was being undertaken) to participate in focus group discussions. Key informants for the study were: Community Health Extension Workers (CHEWs), supervisors of the CHWs; heads of health facilities which the CHWs are affiliated with; Clinicians within these health facilities; Public Health Officers; and District and County Medical Officers of Health.

### Study design

This study employed a qualitative design using a semi-structured questionnaire for in-depth interviews and focus group discussions (FGDs). CHWs were conveniently sampled from two CUs in the selected study sites. A pilot study was first conducted in a community unit in the Kisumu West district. The questionnaire captured questions on the perception of mothers/guardians of under-five children and other key informants on CCMm as well as the appropriateness and attitude of the CHWs who deliver CCMm, among others. The interview with the CHWs focused on their perception of the work and attitude of others towards them. The study was conducted from May to September 2014.

### Sample size

Ten (10) CHWs who had been trained on CCMm from each community unit was selected for the FGDs. Ten (10) mothers/guardians of children under the age of five years who had enjoyed the services of the CHWs in the previous year, were also selected from each community unit. In all, 100 community members were selected from the ten community units within the five districts for the study.

Key informant interviews were conducted with CHEWs who supervise the CHWs within each community unit. In each community unit, the Clinician-in-charge of the health facility affiliated with the CCMm program was chosen for the key informant interviews. This came to a total of ten (10) CHEWs and ten (10) clinicians as key informants. For each of the five districts, the public health officer (PHO) was also interviewed as a key informant.

### Data collection

The interview guide for the study was pre-tested in a community unit in the Kisumu West sub-county. The FGDs and key informant interviews were to assess the perception of the different categories of people interviewed. The questions for the FGDs and interviews were however different depending on who is being interviewed in order to capture different themes and to answer different questions. The questionnaires for the FGDs were first written in English, and then translated into the local language (Dholuo or Swahili). It was later back-translated into English by another person to ensure accuracy.

FGDs were conducted at health facilities for mothers/caregivers of under-five children conveniently sampled from households within the same CU who had been served by a CHW. FGDs for the CHWs were held at a central point accessible from affiliated health facilities. The CHEWs were interviewed individually as key informants at the health facility.

The remaining key informants such as heads of health facilities, clinicians, Public Health Officers and District and County Medical Officers of Health were also interviewed using semi-structured questionnaires. The questionnaire centered on assessment of the needs of CHWs to undertake CCMm. All study participants signed a consent form after an explanation of the study purpose.

### Data management and analysis

All the interviews were audio-recorded, transcribed, translated and de-identified. The transcripts were read as individual wholes to achieve an overarching understanding of the data. The data was coded and finally organized into themes based on the answers of the participants using Nvivo version 7 software. The assessment of the needs of the CHWs was explored based on a common induction approach to coding based on phenomenology rather than pre-existing theory [13]. The results of the coding were compared to ensure consistency of text segmentation and the application of codes. Periodic checks were carried out to ensure intercoder agreement.

## Results

### Demographic characteristics of respondents

The mothers that were interviewed in the FGDs were between 19 and 36 years with a median age of 24 years. The ages of CHWs interviewed ranged from 25 to 59 years of age with a median age of 32. CHEWs were aged between 32 and 47 years with a median age of 37, while the ages of the clinicians and district/county director of health ranged from 28 to 52 years. Whilst most (56 %) of the CHWs were females, all of the CHEWs were males. Among the clinicians and PHOs that were interviewed, 40 % (8/20) were female. In terms of educational level, majority of the CHWs (64 %) had finished secondary school, the rest only having finished primary education. With the mothers, only 22 % had finished secondary school, with 54 % completing primary education while 24 % had no education. None of the participants declined to be part of the study.

## Perception of CHWs on their needs

### Working materials

In assessing needs that facilitate effective services for malaria diagnoses and treatment in the community, the CHWs alluded to provision of the basic essential supplies being the most important. These included: drugs for treating clients who are sick of malaria; pain killers; gloves; rapid diagnostic test kits (RDTs); cotton wool and ethanol; bins to dispose of RDTs; and lancets for pricking clients. They asserted that RDTs could sometimes run out for weeks with the health facility alluding to none being available due to stock-outs. Whilst some CHWs from particular CUs asserted that they had never been given drugs since their training, others indicated they were given drugs immediately after their training but these were not replenished when they ran out. The only option therefore, they opined, was to keep referring all clients who test positive to the health facility. None of the CHWs reported having been given items like ethanol to clean the finger before pricking the clients or dustbins to dispose of rubbish after a session.

On logistical support to enhance their work, the CHWs mentioned gumboots as necessary tools required on rainy days and/or to serve muddy communities. All of the CHWs asserted that accessing a client’s house could be difficult when it rains since it becomes muddy and difficult to walk through the mud without a gumboot. Other logistical supplies mentioned included bicycles or motorbikes for movement to distant areas to visit clients as well as uniforms and ID cards for identification. In reality, each CHW is assigned one hundred (100) households to work with. This consists of approximately 500 people. These households could be located in 1–3 villages. Also, the households are far apart and not clustered together. They all also intimated that access to a mobile phone for their use would facilitate clients reaching out to them and vice versa. CHWs reported on the difficulty clients faced when they needed to talk to them or reach them at night. They asserted that clients sometimes had to send someone to walk to their house to call them when there were issues.

“We need the drugs and the RDTs. We also need raincoats, gumboot and uniforms, at the same time we can say that we need bicycles to help us in moving around.“

CHW from FGD in Chemelil, Kisumu County

“We asked for even gumboots since we walk in muddy areas, but they did not bring them.“

CHW from FGD in Ojolla B, Kisumu County.

### Training

The CHWs perceived other needs to be training/updates on malaria management and therapies to enable them provide better support to clients. They referred to a three-day training on “malaria diagnosis using RDTs and how to dispense anti-malarial drugs to clients” as being the only training intervention received. The CHWs said that a local NGO facilitated this training in collaboration with the Ministry of Health. This training had been done three years prior to the time of the interview. The training was followed up with a week’s attachment at the laboratory of a health facility. The criterion for qualification was the person being able to diagnose a case in the lab correctly. They asserted that no refresher course/training on malaria diagnosis, treatment and prevention practices had been undertaken since then. The CHWs recommended that more NGOs should be encouraged to help in providing trainings and that the government could also run some refresher trainings every now and then.

Before their training on malaria management, they had previously been trained on Community Health Strategy (CHS) by another NGO at the time of recruitment. This was a week-long training. The CHS concept has been described above in this report.B

“….we need refresher training on malaria and maybe something on nutrition. Because if we go out there, we find that the children are malnourished and they are also sick of malaria so when you have knowledge about nutrition you will advise them on how to go about this.“

CHW from FGD in Kenyanya, Kisii.

### Remunerations

A pertinent need raised by the CHWs was the issue of adequate remuneration. They reported that they receive a monthly stipend of Kenya shillings (Ksh) 2000 (USD 22, at the time of interview) which was provided by an NGO which partners with the Ministry of Health. Per their assertion, this is not only for the services they provided on malaria but also for the general services they offered to their clients. They are of the opinion that this amount was not commensurate with their efforts and the time spent going around the village to care for sick people. Coupled with this inadequacy was an inconsistency in the disbursement of the funds. There were complaints that sometimes this could be delayed for several months. The stipend, they intimated was needed to enable them to meet basic needs and as compensation for the time used to support their clients. Compensation for their time afforded them the ability to engage others to assist them in their farms while they were out to offer services to clients. They were of the opinion that since they are part of the health care system, they should be recognized as such and be paid appropriately. Pressed further on how much they perceive to be adequate compensation for their services, the CHWs indicated the minimum amount they should be paid should be Kenyan shillings 10,000 (USD 113) per month. They also recommended a scheme where they could be mobilized and given some piece of land to farm on as a cooperative so that they could get some extra income from this venture.

“…we are sometimes called at night, and you have to leave everything and go. You stop working on your farm so that you can see clients every morning, we also need to be given something reasonable.“

CHW from FGD in Kisii.

“…. we need to be given the materials that can enable us to work, another thing is that money they give us, it should be something that can sustain us and should be on time too. The minimum should be about … Ksh. 10, 000”.

### Recognition from staff of health facilities

The CHWs further complained that they do not receive the necessary recognition from some healthcare workers which tend to undermine their efforts and the CCMm programme. They indicated that sometimes when they take a client already tested and found to be positive for malaria by their system to a health facility, the clinician still insisted on the client being tested again from the laboratory of the health facility. As a result, some clients lose faith and interest in their services. Again, some of them asserted that some technicians at some of the health facilities specifically tell clients not to trust the results of CHWs because they do not have the requisite training.

## Perception of key informants (CHEWs, Clinician Health facility in-charges and PHOs) on CHWs’ needs

### Training

The health workers perceived the needs of the CHWs to be additional/ refresher trainings on the work that they are engaged in. The clinicians asserted that the three-day malaria training given to CHWs was not adequate to empower them to perform at an excellent level and that, each year, they should have the chance to update themselves with new regimen for testing, general diagnosis, and treatment of malaria cases, as is done for clinicians and technicians in the health facilities. One clinician asserted that sometimes when the malaria parasitaemia is very low, the RDT might not pick up the parasites to show positivity in the reading, although the person might from the signs and symptoms be diagnosed with malaria f. She asserted that a CHW might not know this if they are not given refresher courses and might just give antibiotics when the person actually has clinical malaria. The key informants think that since the CHWs are part of the health care system, the government should take up their training and not leave it to NGOs. This, they say, is more sustainable than if it is left with NGOs whose funding might run out at some point.

“…….they need more training and the government must step in to offer this or they should be told to stop and tell the people to come to the hospital. But in my opinion, they should be trained since their work is very important in the communities.“

Health facility in Charge in a KII at Chemelil.

### Incentives

The key informants unanimously alluded to the need to have the CHWs adequately remunerated to retain their services in order to sustain the CCMm. One clinician particularly suggested that the government should go beyond branding CHWs as volunteers (and not remunerating them accordingly), and consider putting them on its payroll to assure CHWs of regular income, no matter how small the funds may be. The CHEWs also think the best way to remunerate the CHW is to support them to establish income generating activities (IGAs), such as small-scale farming where the proceeds will go to the CHWs. They stressed that this IGA idea is being tested in a few community units to see its viability. Also, the CHEWs think that the government could incentivize the CHWs by offering their families and themselves free medical care.

“In my opinion, according to the work load they do, the two thousand they are given is small, they should be given four thousand”. CHEW in a KII.

The best way to motivate the CHWs is to bring them together and initiate an income generating activity for them.“ CHEW in a KII.

“You know they are volunteers, they are working without pay and at least they work. The time has reached for them to be motivated better, because as you know work without motivation is not effective.“

PHO in a KII.

### Working materials

The key informants likewise recognized the need for CHWs to be given some working materials such as: means of transport to reach their clients; torchlight to be used when it is dark; and raincoat or umbrella when it is raining. The clinicians think that CHWs should be given bicycles to facilitate them in moving around. CHEWs reported that sometimes clients of CHWs call them at night and since it is dark and most villages do not have electricity (and, therefore, street lights), CHWs need to have a torchlight to aid them to reach the client’s place. They also confirmed that CHWs had to walk through the rain sometimes to reach the health facility with a client or to see a client, and that provision of a raincoat or an umbrella will do them good. Some of the CHEWs also mentioned that the CHWs could be given a simple mobile phone that their clients could reach out to them on when they need their services, especially at night. A CHEW mentioned that since CHWs walk from house to house, they need to be given an identity card or wear a particular uniform or T-shirt to identify them as people may perceive them to be thieves walking around in the community otherwise.

## **Perception of caregivers of children under 5 years of age and pregnant women**

The community members that included pregnant women advocated that every effort should be made to empower the CHWs to carry out their work. This they said could include training and/or other support similar to ones given to clinicians.

“They are doing a good work and for me personally they are helping me and so when someone can be taken for training or given some money or items to help them in their work so that they can even take care of the children and ourselves, just as nurses do then that should be done to help them.“ Expectant mother from an FGD in Kenyanya, Kisii district.

## Discussion

CHWs represent an important health resource with great potential for providing and extending basic healthcare to underserved populations [4]. In the present study, the needs of CHWs undertaking CCMm in malaria endemic districts in Kenya were assessed in a qualitative study. This is particularly important as their needs will likely affect their performance and job satisfaction. Their needs from the perception of other stakeholders such as healthcare workers and mothers with children under five would provide a holistic view to help shape and promote efficient delivery of CCMm.

In this study, it was found that the CHWs perceived their working materials as their primary needs for them to undertake services for malaria diagnoses and treatment in the community. Without RDTs and ACT, the CHWs will not be able to work, yet most of the workers asserted they usually run out of RDTs and drugs for weeks. This seems a common phenomenon in sub-Saharan African countries [14–16]. All the CHWs interviewed reported that they had never received ethanol rub or even dustbins to dispose of rubbish. It is imperative this need is addressed; the ethanol will make their work safe and could prevent cross-infection. The dust bins on the other hand would ensure a more efficient disposal and prevent needle pricks from used RDTs.

Another area of concern to the CHWs was the lack of recognition, respect and negative attitude of some staff of health facilities. The fact that some of the healthcare workers do not appreciate their services in the communities can affect the zeal and willingness to operate as voluntary health workers in the communities. The CCMm system should involve the clinicians so that the CHWs are well incorporated into the system for effective management of malaria in communities. The respect and support from qualified health workers and the health system is essential to the success and retention of health volunteers [4, 16–18].

Again, other logistical needs mentioned by CHWs and CHEWS, key informants as well as clinicians included gumboots, bicycles or motorbikes, mobile phones, torchlight, raincoats, uniforms and ID cards for identification. These non-monetary incentives mentioned by the participants have also been suggested in similar studies as ways of sustaining CCMm [6, 19]. The gumboots, raincoats, bicycles or motorbikes will enable them to reach community members even when it is raining. Also, the mobile phones will improve effective communication between the community members and the CHWs. The uniforms and ID cards will enable the community members to readily recognize them as health workers in the communities [20] and not mistake them for intruders. This will give them some recognition and prestige in the communities and subsequently serve as a source of motivation.

The CHWs, pregnant women and key informants expressed the need of training and updating the CHWs on malaria management as changes (e.g. to treatment) might have cropped up since the inception of the CCMm or supposed training of the CHWs. The key informants on the other hand thought the trainings should not only be left to the NGOs alone, but that the government should also take it up since the CHWs are also part of the healthcare system in Kenya.

The CHWs are part of the health system but are not on government payroll like the other formally-trained personnel. They, therefore, receive an allowance but not a salary with benefits such as pension and health insurance. Although the CHWs are considered volunteers, they revealed that the Ksh. 2000 (USD 22) they received from an NGO was grossly inadequate for the services they provide the community members including CCMm. The result of this study confirms previous findings of variations in incentives in low- and middle-income countries. These incentives varied from monetary incentives such as reasonable payment to nonmonetary incentives like community recognition, and acquisition of value skills as reported by Ormel et al. [21]. A study in Tanzania also found that CHWs are motivated to volunteer to work and apply knowledge gained to their own problems and those of their families and communities [22]. The CHWs mentioned a minimum amount of Ksh. 10,000 (USD 113) as a financial incentive that will compensate them for their time and also help them engage others to assist them on their farm as they attend to community members. To sustain CCMm, remuneration will be needed to compensate CHWs as volunteerism cannot be relied on entirely to sustain the program [21].

In order for the volunteers to remain focused on their duties in promptly managing malaria in communities, efforts should be made to support them on their farms. Some earlier studies have revealed volunteers who do not get support from community members, pertaining to their faming activities are more likely to drop out of their volunteer work and concentrate on the farming [23] as that is their source of livelihood.

## Conclusions

CHWs undertaking CCMm have a valuable role to play in controlling malaria in developing countries such as Kenya. However, their needs such as logistical supplies, means of transportation, incentives and recognition from health care professionals must be met. These needs must be addressed by the government through the ministry of health, national malaria control programmes, civil society organizations (that are working on malaria prevention and control) in order to motivate and retain them in efficient delivery of services in communities. This study adds to the evidence that the CHWs together with other stakeholders perceive monetary as well as non-monetary incentives as crucial to their services. The gaps in requisite needs identified including refresher training programs must be taken into consideration for the efficient delivery of CCMm in communities.

## Data Availability

Data for the manuscript are available in the Kenya Medical Research data repository to anyone who might need it.

## Abbreviations

ACT: Artemisinin-based combination therapy
CCMm: Community Case Management of malaria
CHEWs: Community Health Extension Workers
CHS: Community Health Strategy
CHWs: Community Health Workers
CU: Community Units
FGDs: Focus group discussions
IGAs: Income generating activities
KEMRI: Kenya Medical Research Institute
KII: Key informant interview
Ksh: Kenyan shillings
NGO: Non-governmental organization
PHO: Public health officer
RDTs: Rapid diagnostic tests
